# “The Graying Minority”: Lived Experiences and Psychosocial Challenges of Older Transgender Adults During the COVID-19 Pandemic in India, A Qualitative Exploration

**DOI:** 10.3389/fpsyt.2020.604472

**Published:** 2021-01-08

**Authors:** Debanjan Banerjee, T. S. Sathyanarayana Rao

**Affiliations:** ^1^Department of Psychiatry, National Institute of Mental Health and Neurosciences (NIMHANS), Bengaluru, India; ^2^Department of Psychiatry, JSS Medical College and Hospital, JSS Academy of Higher Education and Research, Mysore, India

**Keywords:** COVID-19, coronavirus, pandemic, older adults, transgender, gender minorities, lived experiences, qualitative

## Abstract

**Background:** The Coronavirus disease 2019 (COVID-19) has emerged as a global health threat. Certain factors like age, an immunocompromised state, and social impoverishment, etc. can add to health vulnerabilities during this pandemic. One such group is older transgender adults, who often bear a combination of these risks. As the world is aging fast, their numbers have also been increasing. With this in mind, this study explores the lived experiences and psychosocial challenges of older transgender adults during the COVID-19 pandemic in India.

**Methods:** A qualitative approach was used. Ten individuals with “transgender” identity above the age of 60 were recruited with consent through purposive sampling. In-depth interviews were conducted on the telephone using a pre-designed interview schedule. They were recorded, translated, and transcribed verbatim. Hasse's adaptation of Colaizzi's phenomenological method was used for analysis. Independent coding and respondent validation were used to ensure the rigor of data.

**Results:** The super-arching categories (with themes) were marginalization (“second” priority, stigma, social disconnection), the dual burden of “age” and “gender” (ageism, othering, and psychosexual difficulties), and multi-faceted survival threats (physical, emotional, financial) during the pandemic. Social rituals, spirituality, hope, and acceptance of “gender dissonance” emerged as the main coping factors, whereas their unmet needs were social inclusion, awareness related to COVID-19, mental health care, and audience to their distress.

**Conclusion:** The elderly gender minorities are at increased emotional and social risks during the ongoing pandemic, and their voices are mostly unheard. The need for policy implementation and community awareness about their social welfare is vital to improving their health and well-being.

## Introduction

The last 8 months have seen the emergence of a new global health threat, the Coronavirus disease 2019 (COVID-19). After being declared a pandemic by the World Health Organization (WHO), it has affected more than 37 million, with nearly a million people succumbing to the infection (1). After a four-phased lockdown, India has faced a surge of cases and is presently among the countries with the highest case burden (1). Every section of the population has been facing unique challenges during the outbreak but certain minorities are at increased risk in terms of the direct effects of the virus, its psychosocial offshoots, and the lockdown and distancing measures that are used to attempt to contain it. Age and immunocompromised states have been documented to be the two most important factors in deciding the morbidity and fatality rate of COVID-19 (2). The elderly have a unique bio-psychosocial vulnerability. It includes increased pulmonary involvement due to the virus, risk of psychological disorders like depression, anxiety, sleep disturbances, and social factors like loss of autonomy, loneliness, and isolation (3). Certain minority sections have a combination of many such risk factors, one of them being the older transgender population (as a part of the Lesbian, Gay, Bisexual, Transgender, and Queer–LGBTQ community). They share the combined risks of all the issues mentioned above and are primarily neglected in disaster preparedness and management planning. Besides, victimization from the traditional social stereotyping, “third gender” based discrimination, and associated factors like poverty and administrative apathy, they also have increased dependency and segregation based on age. As a part of the Movement Advancement Project, a recent brief by the Centre for American Progress discussed that there are 2.7 and 1.1 million people of the LGBTQ community above the age of 50 and 65 years, respectively. Within this, 20 percent of them are “people of color,” which further worsens health disparities during COVID-19 (4). Data also showed that older transgender adults suffer from mistreatment at long-term facilities and that they have double the risk of poverty and social impoverishment (5). Many transgender individuals also remain on gender-affirming (hormonal or surgical) treatments that have been shown to improve their quality of life, especially in older adults (6), and access to and the availability of such treatments might be a challenge during disaster situations, leading to unforeseen physical and emotional consequences.

The World Professional Association for Transgender Health (WPATH), in association with the Sappho Good Practice Guide, India, has laid down consensus guidelines for diagnosis, hormone therapy, recommendations for sex reaffirmation surgeries, and subsequent follow-ups. The guidelines stress multi-disciplinary efforts, appropriate knowledge regarding the procedures, and adequate psychological support, both pre and post-treatments (7). However, prevalent misconceptions and misinformation in India have led to unscrupulous “conversion therapies” that are unfortunately recommended to “cure” transgender and homosexual individuals. Mostly practiced by faith-healers, preachers, shamans, and quacks, these “curative treatments” commonly involve unsupervised steroids and sex reassignment surgeries (SRS) without consent and the understanding of the individuals involved, which can be psychologically devastating. Although there is no specific Indian law prohibiting “conversion therapy,” it has been proposed that it violates the Right to Privacy (Article 21 of the Indian Constitution) and has been widely regarded as “illegal and unethical” by the Indian Psychiatric Society (IPS) (8). A positive step in this regard has been the Transgender Person (Protection of Rights) Act, 2019, based on “equitable access to health” for this special population. Under this Act, the government provisions for accommodation and education for transgender persons and there is a mandate that at least one Government hospital in every state needs to provide SRS free of cost, with informed consent and counseling. The exact rules are expected to be further clarified and implemented soon, but whether it improves the “rights” of transgender individuals remains to be seen (9).

In India, transgender people are traditionally known as “*Hijras*.” They are often equated with “*Kinnars”* (mythological singers and dancers), as represented in the Kamasutra (ancient Hindu text of sexuality) and even in the epics like Mahabharata (in characters such as *Brihanalla* and *Shikhandi*) (10). For generations throughout history, they have undergone poverty, rejection, neglect, and separation from their own families due to their “identity.” Some even undergo rituals (Nirvaan) to remove their genitalia (11). Over time, their communities have become well-organized, claiming their rights. Irrespective of the widespread advocacy in popular media and literature, they have been subject to socio-economic neglect for decades and legal ambiguity about their sexual identity (12). Even though the Supreme Court in 2014 recognized the Hijras as the “third gender” and subsequently in 2018, decriminalized Section 377, which stated consensual sexual activity between adults of same-sex as a crime, the social acceptance of these legislations is far from reality, and the discrimination against these gender minorities continues (13). Literature related to their “own stories” is scarce, especially in the aged population.

## Methods

### Study Design and Participants

The study followed a qualitative method, using a social constructivist paradigm. In contrast to the positivist paradigm, this paradigm permits the researcher to be open-minded and flexible in exploration, rather than intervening or analyzing based on pre-fixed notions. Furthermore, we chose the phenomenological approach as we wanted to understand the specific “experiences and challenges” of a particular population in the context of an ongoing crisis. Such paradigms have been used in previous studies for studying phenomena like experiences of motherhood, pain, and post-traumatic stress among women, war veterans, and disaster-survivors, respectively (14). Telephonic interviews were conducted with 10 elderly people (aged above 60 years) from the LGBTQ community, who identified their gender identity as “transgender.” We considered the age of “60” as a cut-off for the elderly in this population based on the United Nations/WHO age recommendations (15). However, a range of people aged between 50 and 65 years has been taken in earlier studies on older LGBTQ adults. As access to this sector of the population is difficult, we used purposive sampling. The index participant was known to one of the researchers, who eventually introduced them to interested others. We tried to obtain detailed descriptions of their experiences and the challenges they have faced during the COVID-19 pandemic and the associated lockdowns. A phenomenological approach was used for analysis.

### The Working Definition of “Transgender”

Though transgender or “trans” is commonly used as an umbrella term, for this study, we considered “transgender” as any person for whom their gender identity or expression is different from the sex assigned at birth (or that written in their birth certificate) (16).

We obtained appropriate ethical approval from the Institute board. The participants were initially contacted via telephone, informed about the objectives of the study, and we sought informed consent verbally. Interestingly, all 10 participants welcomed the study initiative and were willing to participate without hesitation. A General Health Questionnaire (GHQ)-12 and Hindi Mental Status Examination (HMSE) were used as screeners for any psychiatric and cognitive disorders. The cut-offs were 3 and 19, respectively (17, 18). We obtained thematic saturation with seven participants. However, we interviewed three more for super-saturation of the data. To ensure confidentiality, we assigned respective numbers (instead of names) to maintain anonymity in transcripts. All transcripts were audio-recorded with consent and then transcribed verbatim. Only the researchers had access to data, which was password protected. The study followed the Standards for Reporting Qualitative Research guidelines (19).

### Procedures

The initial semi-structured interview schedule was designed based on a literature review, which was later modified based on the first two interviews. In that sense, they can be considered to be a pilot for this study. We recorded the socio-demographic details in a separate datasheet. The questions involved in the schedule were open-ended, facilitating rich data regarding their difficulties during the COVID-19 pandemic, their psychosocial needs, access to health care, perceived stigma and discrimination, and the effects of lockdown measures. The salient questions of the interview schedule are summarized in Appendix.

Probing questions like “*can you tell me more about it,” “how did that happen,” “please elaborate on the context,”* etc. were used to receive rich data on their lived experiences. The analysis was done simultaneously with data collection by both the researchers independently to add to the rigor.

### Data Analysis

We used Haase's adaptation of Colaizzi's method for analysis (20, 21). It adopted a phenomenological model, which involves the exploration of the subjective experience under investigation. This approach was chosen as we wanted to know the “lived experiences” of the transgender population. The concept of “intersubjectivity” was used in the analysis to understand the circumstances of “social suffering” from the viewpoint of these individuals. Colaizzi's method essentially involves the following sequential steps:

- Familiarization (running through the transcript several times for a better understanding).
- Identification of significant statements and restating them in “general” terms.
- Formulating meanings relevant to the phenomenon of interest.
- Clustering “identified meanings” into categories, themes, and sub-themes.
- Rigorous discussion among researchers to develop an exhaustive description of the clustered themes.
- Developing a conceptual structure of the studied phenomenon.
- Respondent validation from the participants (seeking verification of the developed structure).

As mentioned, the verbatim transcripts were translated into English (with cross-translation to check for validity). The transcripts and memos were read several times through thematic coding until significant recurrent phrases emerged, and they were re-described in general terms to formulate contextual meanings and then organized and structured by discussion among the researchers. We clustered the super-arched topics into relevant categories and themes, along with verbatim data supporting each of them.

### Ensuring Scientific Rigor

Trustworthiness and credibility are used to establish rigor in qualitative studies, which are different from the concepts of reliability and validity that underpin quantitative research (22). The researchers independently analyzed data using Colaizzi's methods, as mentioned above, categorizing the themes based on the contextual factors affecting the participants, which were then discussed among the research team to reach a consensus on the structural organization of the results. After the first round of analysis, the researchers went back to the participants, five of whom were interviewed again as part of the process of “respondent validation.” Based on their inputs, the hierarchy of the data was modified and supplemented by this additional information. A data trail was maintained so that the steps of qualitative analysis could be traced back to the original interviews.

## Results

The sample consisted of 10 elderly people from the transgender community. Four participants identified as “third gender,” while two preferred “male” gender, one preferred female, and one did not want to disclose gender and orientation. Six participants resided with their families or friends, and four lived alone. Among the latter, one resided at an old-age care home and another in temporary shelters, frequently living on the streets. They belonged to various states of Karnataka, a mix of lower and middle socioeconomic status. Throughout the pandemic, eight of them resided in the same place, whereas two had changed residence. One of these two included the homeless individual who kept changing temporary shelters for support. Only two participants received an old-age pension, as they had previously been employed in Government sector jobs. Six others were not aware of the senior citizen benefits of the country. Two were unemployed, whereas others did menial jobs for a living, apart from the homeless person, who at times begged at street signals. Four of them did not have a valid VOTER or AADHAR card (proof of identity in India). The mean age of the participants was 66.4 years. The mean GHQ-12 and HMSE scores were 1.7 and 25.2, respectively. The mean duration of the interviews was 45.20 min. We conducted the interviews in April and May 2020 when India underwent a four-phased lockdown to curtail the COVID-19 outbreak. The socio-demographic parameters for each of the elderly participants are mentioned in Table 1.

**Table 1:** The socio-demographics of participants.

**Participant**	**Age**	**Age at transition**	**Socio-economic status**	**Sexual orientation**	**Living arrangement**	**Education**	**Job**	**Old-age pension**
P1	64	18	Low	Bisexual	With son	Not formally educated	Unemployed	No
P2	60	22	Low	Gay	Alone	Class 10	Works in a shop	No
P3	67	Doesn't recall	Middle	Lesbian	Living with a partner	Graduate	Retired	Yes
P4	63	20	Low	Lesbian	With daughter	Class 4	Domestic help	No
P5	70	Doesn't recall	Middle	Queer	In old-age home	Class 6	Unemployed	No
P6	75	Doesn't recall	Low	Gay	With friends	Not formally educated	Vegetable vendor	No
P7	60	28	Middle	Bisexual	With partner	Class 12	Private company	No
P8	69	23	Low	Lesbian	Temporary shelters	Not formally educated	Begging	No
P9	65	Doesn't recall	Middle	Bisexual	Joint family	Graduate	Retired	Yes
P10	71	30	Low	Didn't disclose	Alone	Class 8	Manual labor	No

The experiences of participants during the pandemic were broadly categorized into feelings of marginalization (perceived stigma, discrimination, social exclusion, loss of dignity, and reduced access to health-care), vulnerability due to the “dual burden” of age and gender (prejudice of ageism, impaired sexual well-being, feelings of “othering”), as well as multiple physiological, psychosocial, and economic survival threats. Social rituals and festivities within their community, acceptance of their “gender dissonance,” and spirituality provided them hope and helped them cope with the adversities. Knowledge-attitude-practice (KAP) gap regarding the outbreak was a major concern among them, while the predominant “unmet needs” were social inclusion, emotional well-being, social benefits, and receiving an “outlet or audience” for their suffering.

The resultant super-arching categories and themes, along with the respective sub-themes, are summarized in Table 2. They are supported by the key verbal excerpts of the participants, which are mentioned in the table and contextualized in subsequent discussion.

**Table 2:** Categories, themes, and verbal excerpts of the analysis.

**Super-arching category**	**Subordinate themes**	**Verbal excerpts from participants**
Marginalization	“Second priority” in health care	*“Be it medicine shops or checkups at clinics, the moment they saw me they told me to wait or come later.”* (P2)
	Stigma & discrimination (including self-stigma)	*“I have faced this throughout life. Now people look at me in a way as if I am responsible for the virus.”* *“Masks help not only against the virus Saheb but also to protect my identity.”*
	Social disconnection from peers	*“Amidst all these fears of infection, I wish we could meet and spent some time singing as we used to. But can't travel during the lockdown.”*
	Perceived loss of dignity	*“People around have never really liked me. But now they ridicule me…”* *“Who likes to be treated as an object. I am simply dictated terms by the police on the road and my old-age home manager.”*
Dual burden of “Age and Gender”	Prominence of ageism	*“I hear things like… you people won't change even when you are old! Why Saheb, what have we done!”* *“My family and neighbors don't want to come near me as I am old and probably have got more of the infection…”*
	Deprived psychosexual needs	*“Intimacy with my partner is a major comfort. But he goes for work. So, we don't feel comfortable making love…”*
	“Cornered” in their communities	*“I am mostly not able to join the group due to age. Now even when they discuss any health-related matters among themselves, I am left out.”*
Multi-faceted “Survival threats”	Physiological	*“When they distributed masks, they did not give me. I already stay in an overcrowded room with three others.”* *“I wanted to get tested, but they told me to get HIV testing!”*
	Psychological	*“I feel really lonely and sad. My partner is far away. And people don't talk to me over here.”*
	Financial	*“I had a small job in a shop. That has been closed due to lockdown. No one wants to offer me any financial help. I don't want to resort to begging like others.”* *“The Baadhaai (baby-showering) ceremonies are our main source of bread. We are not being allowed anywhere.”*
Coping	Social rituals and pride celebrations	*“I know about this place where all of us gather and celebrate. Someone told me it is this time of the year. I wish it could have happened.”* *“I am looking forward to the online Pride festivals. It will give me a lot of support in this lockdown.”*
	Acceptance of the discomfort of belonging to the “third gender”	*“What the pandemic taught me was that the virus doesn't discriminate. I finally will at peace with my identity.”*
	Spirituality	*“My prayers and faith in God kept me moving all these days, even when I had to beg for a living. Never knew it will be so helpful now!”*
	Hope	*“I have seen worst times, Saheb. I am hopeful this, too, shall pass!”* *“I believe in tomorrow. The rest of my days, I want to live with that belief. It helps in this uncertainty.”*
Unmet needs	Knowledge, attitude, practice (KAP) related to COVID-19	*“I only know that old people are dying. Do I need to get tested? Some medicine is being recommended. Should I take it?”* *“Someone was explaining something in the local language in a meeting about COVID. They didn't let me in!”*
	Social inclusion	*“I stay alone. Every day with a fear that I will get the infection and die without treatment. I wish I could share this with others.”*
	Mental health care	*“Every time I have gone to the doctor, people like you when they grow old, these things happen. Nobody asks or understands why I get sad and anxious!”*
	The audience for their “voices”	*“I don't know after how many days, someone asked this Budda (old man), about his difficulties!”* *“You really want to know how we feel, or it is just for your research?”*

## Discussion

### Dual Burden of “Age” and “Gender”: Marginalization

Biological disasters like COVID-19 often hit the most vulnerable in the worst ways. Advanced age and belonging to a gender minority group are both crucial susceptibilities during the pandemic. They contribute to the “minority stress” of marginalized populations like the homeless, migrants, socially impoverished, and especially the Black, Asian, and minority ethnic (BAME) communities, as already postulated during the ongoing outbreak. These include social insecurity, unemployment, experiences of racism, prejudice and xenophobia, lack of social welfare benefits, precarious work, increased risk of infection due to lack of precautionary measures and overcrowding, and most importantly, lack of knowledge and awareness related to the pandemic and its related issues (23, 24).

The study participants revealed a sense of increased “ageism” during the ongoing outbreak, which has also been raised as a potential concern in public health guidance measures for the “elderly” during COVID-19.

“Throughout our lives, we have been ‘observed’ differently, now it's even more as we are old. We even get called names when we ask for help.”

**(P6, on discussing information-seeking about the pandemic)**

Such age-related prejudice and attitudes that consider the “elderly” as a “justified loss” for the sake of younger lives were also documented during the Severe Acute Respiratory Syndrome (SARS) and Ebola outbreaks (25, 26). Age itself becomes an essential factor for the “third gender” as many of the societal and sexually acceptable roles of transgender people tend to get to be affected by age.

In the Indian context, transgender people or *Hijras* take to baby showering ceremonies *(Badhaai)*, which are an essential source of income, and during the pandemic have become scarce. Furthermore, the “sexual vitality” and “auspiciousness” of the Hijras, for which one welcomes them in ceremonies decreases with age, and hence older transgender adults often run out of income (27). Seven participants also looked at such social rituals as their source of coping and connectedness with society.

“We look forward to Badhaais and Varanas (rituals)! This is something we have been doing since we are young. Other people welcome us during these times. It has become our true festival! This disease has taken it away from us!”

**(P4, on how she feels excluded from the society during the pandemic)**

“In spite of all the ‘hate’ for us, society requires us for these ceremonies. Now, with the fear of infection, they don't even allow us near people's houses. Our livelihood and connections both are at risk!”

**(P6, while describing the cessation of rituals during the ongoing crisis)**

Most participants mentioned being the “second priority” for health and legal services, including access to medications, medical protective equipment, and testing.

“For [the] last three months, I have been so used to hearing: ‘people like you should come later, any way you are old, what's the need… just go and stay with your people…’ It hurts you know, it feels we are ‘aliens’ in this world. This adds to my uncertainty…”

**(P8, on help-seeking during COVID-19)**

Indian society has been marked with discriminative social reactions toward transgender people, who are often the subject of ridicule and fun. Conforming to social acceptance, many of them reluctantly assume the “social roles” of sexually seductive behavior with the opposite sex, begging, or petty crimes. Talwar (28) in *The Third Sex and Human Rights* discusses the deprivation of human rights, poverty, and violence inflicted upon this community in India. Besides financial constraints and unemployment, many individuals from the Hijra community are forced to pursue a living through extortion, begging, exhibitionism, and sex work (an activity often socially stereotyped as associated with transgender individuals). The violence directed against transgender people has been widespread and often brutal, and is documented as taking place in their places of residence, prisons, police stations, and other public spaces (29). Boggs et al. (30), in their focus group discussions of 73 older LGBTQ participants, mentioned the intersection of “ageism” and “cisgenderism” as an under-recognized barrier to health, social and legal access. Trans PULSE, an Ontario-wide research study, showed a lack of sensitivity to health care and discrimination in health care access among the transgender population (31). This is more pronounced in a pandemic crisis, as amidst the vulnerability, these participants are also the victims of “othering” that classically forms the “we vs. they” dichotomy. “Othering” is a term used to denote expressions of prejudice based on a “group identity,” in this case the “third gender.” Social stereotypes help to maintaining and perpetuate this group-based inequality and marginalization, thus depriving a certain “group” of their rights and social privileges (32).

“See, here she has come again…I have heard this all my life! Now it has increased, and the moment I go to any shop, people start behaving weird and push away. It's as if… I am the source of infection.”

**(P2, when asked about social reactions during the pandemic)**

“They were supplying free masks and soaps. I couldn't stand in the queue due to the constant ridicule and mockery that I saw in people. At times I feel… getting the infection is better than facing such insult at this age!”

**(P9, discussing the precautionary measures)**

The resultant “minority” stress has been explained in terms of the Health, Stigma, and Discrimination model (33), where facilitators like disasters, societal apathy, and prejudice toward age-related and sexual minorities can eventually led to detrimental psychosocial outcomes during crises (23).

Six participants reported self-stigma, a common finding in the LGBTQ community when they feel guilt about their “sexuality” and social notions that are against them, meaning they are further segregated from the mainstream and adopt their “community customs and rituals” (34). Social attribution theories posit that a constant negative stereotype against a certain individual or group can lead to self-doubt, decreased self-esteem and self-stigma, when that individual or group starts internalizing those “faulty beliefs” and attributes them to their social status (35). This causes further social exclusion and decreased help-seeking, especially during crises, which worsens othering.

“I have always been seen as ‘seductive’ in personal and professional circles; I have no idea why! Now I have come to believe that probably my gender is responsible for this… age changes a lot in you… and there are certain ‘blames’ you cannot take any more at this age.”

**(P10, on how she was blamed for being “provocative” in public)**

“I feel that I am different… I have always felt that way… people passing sexual comments and ridiculing me… that's how we, Hijras live…even in this situation, we need to go out for living…but get threatened by people and police telling we are on ‘business’! I get tired of my ‘appearance’ at times and wish it was different.”

**(P4, when asked about livelihood challenges)**

In the above excerpts, participants mentioned social stereotypes against their communities and how they have internalized these misrepresentations over time. While P10 started “believing” the blame attributed to her gender, P4 wished she could change her appearance for the “sake of society.” Other transgender individuals in our study also mentioned “reduced self-esteem” and developing a “hatred toward themselves” after constantly battling social injustice and prejudice. The self-stigma generated in an already marginalized community can be further detrimental to their ability to cope during the crisis.

Loneliness, social disconnectedness, and depression were reported by 8 out of 10 participants. Major factors that have influenced these feelings include restricted travel, physical distancing, and difficulties in using and accessing technology (in 5 participants). Even though digital connectivity and telemedicine have repeatedly been used during the pandemic, they can be real challenges in lower and middle-income (LAMI) countries like India, where technology still fails to reach the masses, especially in the rural areas and minorities. Furthermore, age, with its sensory and cognitive limitations and frailty, can impair the appropriate use of video-connections with loved ones, which can lead to further loneliness and social segregation. Berger (36), in his classic text “Gay and Gray,” describes cases of homosexual and transgender men whose narratives reveal loneliness, existential crisis, and “age” as an acceptance of the age-old discrimination that they face. The “discomfort with their gender” that some participants experienced during most of their lives due to their “transgender” identity seemed more acceptable during the pandemic, as they navigated these adversities. Some attributed these problems to age, others to wisdom, while others felt that “suffering” due to COVID-19 has made them more resilient.

“All throughout I wished I was like the ‘others’! My gender kept bothering me like a curse from birth. Living through so much of difficult times, it mattered little… Everyone was suffering and dying the same way. I don't know when, but I had stopped wanting to be ‘different’!”

**(P2, while discussing discomfort with the third gender)**

“Sometimes I feel this was needed… I witnessed the death of my friends, colleagues… there is so much suffering all around…I somehow feel stronger, more ready to face the world…”

**(P10, on the effect of the outbreak on coping)**

“Age brings in experiences, wisdom, acceptance, and much more… all my life I have faced hardships, even in daily existence… it's tougher times now, deaths, medicines, hospitalizations all around… with my age anyway I have nothing to lose… so I try to make maximum out of my life now, irrespective of everything… I tell others the same…”

**(P5, when asked about suggestions for others in the old-age home)**

Psychotherapy for the elderly often uses such lived experiences, building upon notions of resilience and post-traumatic growth after disasters/crises, based on existential and humanistic approaches (37). Earlier studies have mentioned age and experience as enabling factors for community inclusion (38). However, our participants felt “othered” in their community, which forms a significant part of the societal stigma that is potentially harmful to coping and mental health.

“They say people who are living on the streets are being targeted for having the infection. I have the additional issue of being a Hijra. They were almost forcibly admitting me to the hospital… even without testing…”

**(P1, when asked about the challenges they face)**

There is also a common myth that the elderly do not have sexual needs, and these ideas were challenged by P4 and P7, who mentioned that they “cannot even experience intimacy to soothe us during difficult times as they (their partners) go out for work, and the risk of infection” is present. The pandemic's effect on psychosexual health and deprivation of “social touch” remains largely unspoken but is critically detrimental to well-being. As COVID-19 is a highly contagious infection, this has led to doubts relating to sexual transmission and a definitive fear of intimacy. Sexual relationships and sexual well-being can be affected, irrespective of age or gender specifications (39).

“Our relationship and closeness have literally formed my core strength in the worst of times. He goes out, so we sleep in separate rooms… the infection has created an emotional wall between us…”

**(P3, on relationships during COVID-19)**

“Intimacy with my partner is a major comfort. But he goes for work. So, we don't feel comfortable making love… It makes me weak and vulnerable!”

**(P7, on how the crisis has affected coping)**

It is important to note here that resilience and strength emerging from their relationships and intimacy served as “viable supports” during the pandemic crisis. This is in contrast to models of psychological resilience being conceptualized as “personal internal attributes” based on Western mainstream psychology schools (40). The authors further emphasized the “social functioning” that can be vital for coping and in developing problem-solving approaches during the crisis. In large-scale social threats like the COVID-19 pandemic, the need for social enmeshment and emotional bonding with their partners fostered love, care, and support, which were reflected in our participants.

### Disasters in the Transgender Elderly as “Survival Threats”

Overall, the above factors together generated physiological, emotional, and financial pressures for participants. Losing priority and stigma kept them at a “backfoot” for health care, their voices unheard, and the uncertainty of the COVID-19 situation was amplified due to the double fear of “aging” and “being deprived.” Many of the participants reported mentioning that “old people are dying fast,” which they encountered in the media, with detailed information about morbidities that added to their fear. Besides, it is essential to understand that frequent comorbidity of Human Immunodeficiency Virus (HIV), diabetes, and other chronic medical conditions, compounded by neglect, often leads to persistent immunocompromised states in transgender people. These, along with age, can form the two most crucial risk factors for morbidity and fatality in COVID-19 (41). Associated mental illness and substance abuse often worsen the situation. Seven of our participants mentioned that their suffering lacked the audience, though the community was superficially concerned. It made them feel “invisible to society,” resonating with “how they have always felt.” Empathy and compassion were not sensed by the people around them, and even financial support was difficult to access. In the absence of employment and social benefits (such as a pension) for most of them, this made the situation even more dire.

“My neighbors would not want to interact with me. I was old, staying alone, and even basic needs were difficult… people knew that, but I hardly even got anyone asking how I am doing…”

**(P2, while discussing the unmet needs)**

“‘Take care’ is the maximum assurance that I have received! Nobody bothered about how I needed to care for myself staying on the streets…Even begging didn't help.”

**(P8, on social indifference to suffering)**

There has been a traditional association between the LGBTQ group and disasters. McKinnon et al. (42) mentioned how the voices of this community were largely under-represented by mainstream media during the Brisbane floods or the Christchurch earthquake of 2011. There was mention of similar marginalization during the Queensland floods (43). The same authors have also written about the “Queer domicide” wherein homelessness was a significant offshoot of natural disasters in gender minorities as the administrative policies that respond to these crises remained neutral or respond to the needs of the perceived status quo (44). The *Higashinihon Dai-Shinsai* (The Great Japan Disaster) of 2011, which began with a tsunami and earthquake, led to a lack of shelters, mass stigma, bullying, violence, and social exclusion for the Japanese LGBTQ community, effects that have been vastly under-represented in the literature (45). Systematic research and policies related to the plight of the LGBTQ population in India during disasters also lack standardization. The present study recommends that LGBT rights are incorporated into and allowed for in disaster ethics and disaster preparedness planning. The literature on this subject also documents that many older transgender adults do not have fixed jobs and are dependent on their families, homes, and communities, which decreases their autonomy and increases their risk of abuse. The homeless individual who took part in this study faces overcrowding, lack of quarantine facilities, and proper shelters, an experience shared by thousands of migrants all across India during the lockdown. Their age, gender, and the social crisis are a “triple blow” to their present condition.

“People usually help seniors, don't they! Am I any different because of my gender? Can't I expect the same help from others who are much younger than me…”

**(P5, on being an “invisible sufferer” during the pandemic)**

“There was no respect for age or humanity… how can I expect help!”

**(P7, while talking about distress)**

While these were their vulnerabilities, we will now discuss how transgender individuals have navigated the crisis and the barriers they face.

### Coping, Resilience, and Barriers to Care

Five participants in this study were aware of the Pride movement and ongoing Pride month. Two knew that there was some celebration scheduled for their community at this point of the year, and three were unaware. Pride celebrations emerged after the Stone Wall riots in 1969, and mark the ongoing protest and expression of the social integrity of the LGBTQ community (46). Around 220 pride festivals have been canceled across the world due to the COVID-19 situation, and even though some organizations held events online, there was limited impact and access (47). In India, elderly participation in the Pride Movement has always been scarce (48), which creates a situation of “seclusion within seclusion.” The participants looked forward to a regular get-together of their community as a way of coping during the ongoing crisis. While most did not identify the festival with a “name,” they were aware of celebrations being canceled due to the pandemic situation. Even local festivities within their community were compromised, which affected their social support. This is a notable reflection of emotional expression in the Indian socio-cultural context. While discussing “gender, depression and emotion,” Davar (49) examines Indian folk stories and contrasts the “collective” emotional expressions of Indians rather than the usual homogenized view of singularity. This collectivism was more prominent in the marginalized sector of the population that we studied.

“I know about this place where all of us gather and celebrate. Someone told me it is this time of the year. I wish it could have happened.”

**(P3, while discussing Pride celebrations during a pandemic)**

“I don't know too much about it and what they call [it]. But in our community, we have small celebrations, cooking, singing, and all… nobody dared to do all these… it will lead to more trouble in society, as such we are always blamed…”

**(P8, when asked about festivities in the community)**

Spirituality and hope emerged as essential themes in seven and five participants, respectively. Ross et al. (38), while studying a group of transgender individuals, mentions that “personal development” and optimism are essential factors in building resilience, even though the study subjects were not older adults. Spirituality and hope for the future play an essential role in coping in our study subjects, forming a part of their “self-identity.”

“I used to go to Hanumanji's temple whenever possible, irrespective of all odds. It really helped. There was so much peace there…”

**(P3, while talking about religious practices)**

“God or not, there is a force I believe in and worship. Can't explain! But that gives me hope…”

**(P10, when asked about spirituality and coping)**

The need to identify oneself with society for perceived self-worth, irrespective of disabilities, formed an essential aspect of the community health care needs of the elderly in a systematic review done by Holm and Severinsson (50). A low level of spirituality has been associated with a poor ability to cope emotionally and higher rates of depression in Indian older adults (51).

All the participants agreed that they lacked awareness about the necessary measures and ongoing situation about the pandemic. Only two followed social media updates, and apart from the numbers projected in the newspapers, they had questions about safety and testing for COVID-19. Three were increasingly dependent on their families while four others worked in shops as manual laborers and domestic help and lost working days during the lockdown. Salary cuts and unemployment have been widespread during the COVID-19 pandemic. Data from the PSB Research group in the United States showed that 30 percent of the LGBTQ community had their working hours reduced and salary decreased, compared to 22 percent of the general population (52). This creates a sense of mistrust and anger at the administration for the participants. Media reports mention increased socio-economic deprivation for the Indian LGBTQ community during the lockdown, a rise in abuse at the hands of their own families, and social harassment, which adds to the pre-existing burden caused by the pandemic (53).

All of the study participants agreed that they felt like an “outcast” even when they repeatedly heard people saying “we are in this together.” Eight of them reported lacking an audience for their problems, which was why they welcomed the interview.

“Thanks to your research that you are asking… I felt good sharing these issues with someone, very few bother… already old, now more of a burden to the society!”

**(P4, toward the end of the interview)**

“Media arrives and raises thousands of questions when needed. Nothing changes! Maybe nothing will, but at least you asked…”

**(P3, while reflecting on the present study)**

Social integrity and support are essential components in creating resilience during widespread disasters. These needs were not met in most participants. To summarize, the “dual burden of ageism and third gender” along with marginalizing factors decreased their access to health care and created physically unsafe and emotionally insecure environments, which along with “dependence” and “poor awareness” increased their physical and psychosocial vulnerabilities to the COVID-19 situation.

Our study had a small sample size and is subject to the usual limitations of qualitative research, such as subjective interpretation and reduced generalizability. The ongoing pandemic crisis could also have exaggerated participant responses to questions related to their suffering. However, in a marginalized sector of the population, each voice matters, and our study benefits from a rigorous methodology, analysis, and reflecting and providing a platform for these usually “unheard and invisible” voices. As requested by the participants, the researchers present these “narratives” as representations and accounts of social suffering, rather than mere data.

## Conclusion

India is aging fast, as are the increasing number of gender minorities. Guidance on the care of the elderly by the WHO as well as the Ministry of Health and Family Welfare (MoHFw) in India are comprehensive, but unfortunately fail to mention the already neglected experiences of the transgender community (54, 55). Wang et al. (41) have recently appealed to the Government and private sector to consider the holistic care of transgender groups, advocating community-based screening for their needs, online consultations, and a reshaping of policies that accommodates their health-care needs and enhances access. Associated with this are HIV management and harm-reduction techniques for substance abuse in this population during the pandemic. *Aging with Pride: The National Health, Aging and Sexuality/Gender* (NHAS) study has proposed the “Health Equity Promotion” model for older transgender adults, based on a bio-psychosocial understanding of their unique vulnerabilities (56). The Coronavirus Preparedness and Response Supplemental Appropriations Act and The Coronavirus Aid, Relief, and Economic Security (CARES) Act passed by the U.S. Congress during the pandemic are inclusive of LGBTQ communities (57). Such models could be adopted by the Indian Government, especially with the numbers of this population increasing. Our study sample was small, but the rich data from the participants, the thematic saturation, and the holistic representation from various backgrounds strengthened the study. These results indicate that the needs of gender minorities are still largely unmet, especially in older adults. However, these findings need to be interpreted in the Indian socio-cultural context. The Indian Pandemic Act of 1897, needs to be overhauled to consider the needs of both seniors and the transgender population, which could help preparedness for similar crises in the future. Banerjee and Nair (23) have discussed the different “vulnerability areas” of transgender individuals during the COVID-19 crisis and suggested interventions to mitigate physiological risks, social discrimination, sexual stigma, substance abuse, and to preserve psychological well-being, economic stability, sexual health, and gender-based equality. The authors highlight the unique needs of the elderly LGBTQ population and foreground the need to prevent ageism, stigma, and appropriate social rehabilitation measures. The under-representation of older adults in India's LGBTQ movements has been a growing concern in recent years, particularly in terms of their civil rights and socio-economic security. Care homes are often not suited to their needs and can turn into potential sites of abuse. Transgender individuals are not immune to the frailty, cognitive, and sensory deficits of aging and will need similar care. This sensitivity needs to be emphasized at all levels, by active collaboration between physicians, human rights activists, the media, and government administration (58). As the pandemic is still in its early period, the coming months will be crucial for undertaking more systematic research into lived experiences and risks due to COVID-19, apart from focusing on testing and symptom-based management. Similarly, our collective responsibility is to be aware of the unmet needs of this community in terms of social inclusion, care, and support rather than discrimination. Only then can their health emerge as a priority and not an option.

## Data Availability Statement

The original contributions presented in the study are included in the article/supplementary materials, further inquiries can be directed to the corresponding author/s.

## Ethics Statement

The studies involving human participants were reviewed and approved by JSS Academy of Higher Education & Research, JSS University, Mysuru, Karnataka, India. The patients/participants provided their written informed consent to participate in this study.

## Author Contributions

DB was involved in data curation, analysis, and drafting of the manuscript. TSSR was responsible for designing the study, data collection, reviewing, and editing the manuscript. Both authors conceived the study, read and agreed on the final version of the paper.

## Conflict of Interest

The authors declare that the research was conducted in the absence of any commercial or financial relationships that could be construed as a potential conflict of interest.

## Appendix

The Key Questions of the Semi-structured Interview Schedule

- What difference did you face between the pre-pandemic and the COVID-19 times?
- How do you think the pandemic has affected your mental well-being?
- How do you think your age affected your perceptions related to the current crisis?
- What challenges did you face due to the pandemic situation?
- What type of support have you received?
- What do you think could have been done to make your experiences better during this time?
- What were the difficulties in seeking psychological care during the pandemic?
- What were your unmet needs?
- How do you think the COVID-19 situation might affect your future?
- What message would you like to provide for the elderly from the same community?
